# Utilizing network pharmacology to explore the underlying mechanism of *Radix Salviae* in diabetic retinopathy

**DOI:** 10.1186/s13020-019-0280-7

**Published:** 2019-12-30

**Authors:** Chun-Li Piao, Jin-Li Luo, De Jin, Cheng Tang, Li Wang, Feng-Mei Lian, Xiao-Lin Tong

**Affiliations:** 1 Institution of Shenzhen Hospital, Guangzhou University of Chinese Medicine (Futian), Shenzhen, 518000 Guangdong China; 2 Institution of Guang’anmen Hospital, China Academy of Chinese Medical Science, Beijing, 100000 China

**Keywords:** *Radix Salviae*, Network pharmacology, Diabetic retinopathy

## Abstract

**Introduction:**

*Radix Salviae* (Dan-shen in pinyin), a classic Chinese herb, has been extensively used to treat diabetic retinopathy in clinical practice in China for many years. However, the pharmacological mechanisms of *Radix Salviae* remain vague. The aim of this study was to decrypt the underlying mechanisms of *Radix Salviae* in the treatment of diabetic retinopathy using a systems pharmacology approach.

**Methods:**

A network pharmacology-based strategy was proposed to elucidate the underlying multi-component, multi-target, and multi-pathway mode of action of *Radix Salviae* against diabetic retinopathy. First, we collected putative targets of *Radix Salviae* based on the Traditional Chinese Medicine System Pharmacology database and a network of the interactions among the putative targets of *Radix Salviae* and known therapeutic targets of diabetic retinopathy was built. Then, two topological parameters, “degree” and “closeness certainty” were calculated to identify the major targets in the network. Furthermore, the major hubs were imported to the Database for Annotation, Visualization and Integrated Discovery to perform a pathway enrichment analysis.

**Results:**

A total of 130 nodes, including 18 putative targets of *Radix Salviae*, were observed to be major hubs in terms of topological importance. The results of pathway enrichment analysis indicated that putative targets of *Radix Salviae* mostly participated in various pathways associated with angiogenesis, protein metabolism, inflammatory response, apoptosis, and cell proliferation. The putative targets of *Radix Salviae* (vascular endothelial growth factor, matrix metalloproteinases, plasminogen, insulin-like growth factor-1, and cyclooxygenase-2) were recognized as active factors involved in the main biological functions of treatment, which implied that these were involved in the underlying mechanisms of *Radix Salviae* on diabetic retinopathy.

**Conclusions:**

*Radix Salviae* could alleviate diabetic retinopathy via the molecular mechanisms predicted by network pharmacology. This research demonstrates that the network pharmacology approach can be an effective tool to reveal the mechanisms of traditional Chinese medicine from a holistic perspective.

## Introduction

Diabetic retinopathy (DR), which results from chronic high blood glucose levels, is one of the most common and serious complications of diabetes mellitus, which is the main cause of adult-acquired blindness [1]. A recent meta-analysis of 243 population-based studies shows that globally, in 2010, out of 32.4 million blind and 191 million visually impaired people, 0.8 million were blind and 3.7 million were visually impaired because of DR [2]. Another meta-analysis, based on 288 studies, concluded that 2.6 million people were visually impaired (blindness and moderate to severe vision impairment) from DR in 2015 and this is estimated to rise to 3.2 million in 2020 [3]. Furthermore, DR leads to a poor quality of life and an increased risk of other diabetes complications and mortality, which brings severe social burden [4, 5]. Currently, DR is mainly treated by laser photocoagulation of the retina, anti-vascular endothelial growth factor (VEGF) drug therapy, hormone therapy, and surgical treatment. However, these treatments might contribute to certain adverse reactions, such as the increase of angiogenesis, rise intraocular pressure, and retinal hemorrhage [6, 7] and the effect of single or combined treatment is limited. Therefore, it is urgent to discover potential therapeutic targets and develop new therapeutic strategies for the treatment of DR.

Traditional Chinese Medicine (TCM) is a comprehensive medicinal system that is characterized by its satisfying therapeutic effects and minor side effects. TCM is widely used in Asian countries, especially China [8]. TCM is characterized by multiple ingredients that have a variety of advantages, such as synergy, reduction of side effects, and improvement of adaptive resistance [9]. TCM network pharmacology not only identifies and optimizes multiple target interventions by modeling signaling pathways and specific processes [10], but also measures the efficacy of drugs, especially multi-target drugs [11]. Recently, TCM network pharmacology has been widely applied to the exploration of complex diseases, such as cancer, renal injury, and heart failure [12–14]. Based on the theory of traditional Chinese herbal medical science, TCM can offer a treatment for the prevention and treatment of DR in a systematic way. *Radix Salviae* is a species of Labiatae that is distributed throughout the country. As a traditional medicinal plant, it has satisfactory drug efficacy for the alleviation of DR, which indicates the existence of certain pharmacological components in *Radix Salviae* [15]. We discovered, in clinical practice, that *Radix Salviae* can effectively relieve the clinical symptoms of DR, such as local visual field defects, vision loss, and visual impairment [16]. However, the pharmacological mechanisms of *Radix Salviae* are still unknown.

With the rapid development of bioinformatics, systems biology, and poly-pharmacology, network pharmacology, based on the concept of “Disease-Gene-Target-Medicine”, can explore the complex mechanisms of medicine on the human body [17]. This is in keeping with the holistic view of TCM and the mechanisms of TCM formulas are multi-ingredient, multi-pathway, and multi-target [18].

**Fig. 1:**
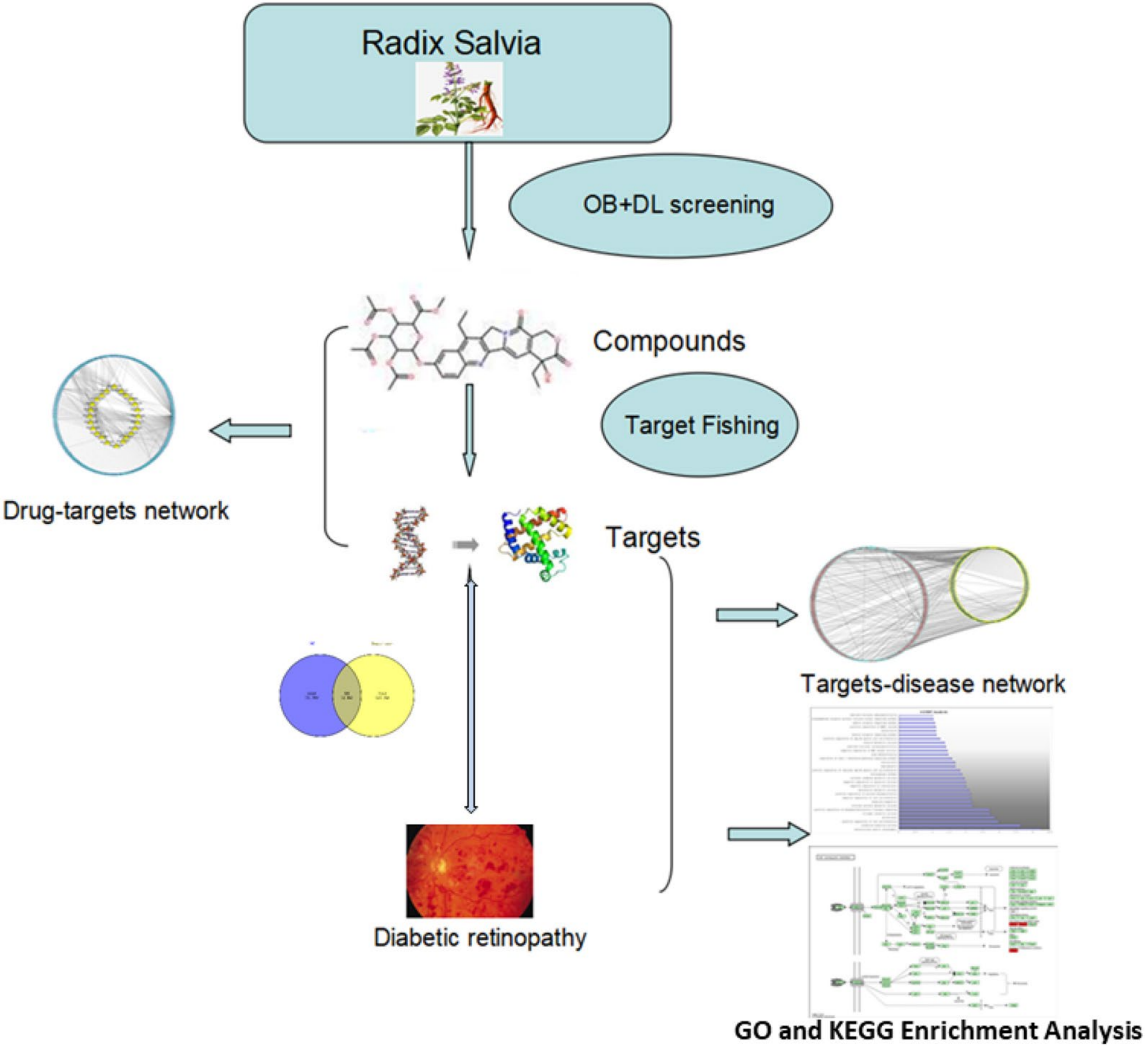
The flowchart of the network pharmacology-based strategy for deciphering the mechanisms of *Radix Salviae* on DR

The aim of our study was to screen the related ingredients of *Radix Salviae* using multiple databases and acquire the potential targets by target fishing. Then, we aimed to screen the related targets of DR by consolidation of a multi-source database. Based on the matching results between *Radix Salviae* potential targets and DR targets, we aimed to build a protein–protein interaction (PPI) network to analyze the interactions among these targets and screen the hub targets based on topology. Moreover, using The Database for Annotation, Visualization and Integrated Discovery (DAVID) bioinformatics resources, we aimed to obtain the enrichment analysis of the Gene Ontology Biological Process (GO-BP) and Kyoto Encyclopedia of Genes and Genomes (KEGG). This study is necessary to investigate how *Radix Salviae* alleviates DR via the molecular mechanisms predicted by network pharmacology and how the network pharmacology approach can be an effective tool to reveal the mechanisms of TCM. The flowchart of the experimental procedures of our study is shown in Fig. 1.

## Methods

### Data preparation

#### Chemical ingredients database building

To collect the ingredients in *Radix Salviae*, we used the Traditional Chinese Medicine System Pharmacology Database [19] (TCMSP, http://lsp.nwu.edu.cn/tcmsp.php, 2019.8.11), a specialized pharmacological platform for TCM. Two hundred and two herbal ingredients were recorded in this process.

### Active ingredients screening

#### Oral bioavailability (OB) prediction

OB is the percentage of an orally administered drug that reaches the systemic circulation. It is one of the most used pharmacokinetic properties in drug screening. In this process, the OB threshold was set as 30% and those ingredients with OB ≥ 30% were selected as the active ingredients for the next step [20].

#### Drug-likeness (DL) evaluation

DL is a molecular parameter that measures the absorption, distribution, metabolism, and excretion of drug molecules affected by their pharmacokinetics. By evaluating prospective “drug-like” compounds, DL optimizes the pharmacokinetic and pharmaceutical properties, such as chemical stability and solubility. The DL level of the compounds was set as 0.18, as this is the selection criterion for “drug-like” compounds in traditional Chinese herbs [21]. In this study, those ingredients with DL ≥ 0.18 were selected.

### Target fishing

The active ingredients of drugs play a role in related biological functions via targets. Our study located targets by target fishing based on the candidate ingredients. Retrieving the small molecular structure information of the active ingredients in *Radix Salviae* on the PubChem database (https://pubchem.ncbi.nlm.nih.gov/), we fished targets with a screening online tool called the Swiss Target Prediction webserver [22] (http://www.swisstargetprediction.ch/index.php).

### Disease targets database building

We collected DR targets from four source databases. The databases used in our study were: the DrugBank database (http://www.drugbank.ca/, version 4.3, 2019.8.11), Online Mendelian Inheritance in Man (OMIM) database [23] (http://www.omim.org/, 2019.8.11), DisGeNET v6 database [24] (http://www.disgenet.org/, version 6.0, 2019.8.11), and Genetic Association Database [25] (https://www.geneticassociationdb.nih.gov/, 2019.8.11).

Finally, we matched the prediction of the targets of *Radix Salviae* active ingredients and the retrieval of the related targets of DR and chose the overlapping targets as the related targets of *Radix Salviae* for the treatment of DR. The targets were then processed by String [26] (https://string-db.org/, 2019.8.13) to draw the data of PPI.

### Network construction

#### Network construction method

(1) Compound-target network (C-T network); (2) *Radix Salviae* target-DR target interactional network (T-T network); (3) Target-pathway network (T-P network). The pathway information of targets was screened from the result of KEGG pathway enrichment. Cytoscape3.6.0 (http://www.cytoscape.org/, 2019.8.14), an open-source software platform for visualizing complex networks and integrating these with any type of attribute data, helped build visualized network graphs [27].

#### Network topological feature set definition

We selected two parameters to evaluate the topological features of every node in the interaction network. “Degree” is defined as the number of links to a node, which reflects the frequency of interaction between a node and other nodes [28]. “Closeness Centrality” measures the mean distance from one node to another. A geodesic path is the shortest path through a network between nodes [29]. The more important the output of a node, the higher the value of this node in the network. Therefore, the levels of the two parameters represent the topological importance of the nodes in the network.

### Enrichment analysis

We used DAVID (https://david.ncifcrf.gov/, v6.8,2019.8.14) [30] for GO enrichment analysis and KEGG (http://www.kegg.jp/, 2019.8.14) [31] for pathway enrichment analysis.

## Results

### Active compounds in *Radix Salviae*

Retrieved from TCMSP, there were 202 related components in the whole formula in total. According to the active ingredient screening thresholds of OB ≥ 30% and DL ≥ 0.18, 65 active ingredients were selected.

### Target prediction and analysis

We conducted target fishing on the 65 active ingredients based on chemical similarity, obtaining 287 related targets. The 65 active compounds that were obtained are listed in Table 1.

**Table 1:** The list of 65 compounds of *Radix Salviae* and their OB and DL

ID	Compound	OB	DL
MOL001601	1,2,5,6-Tetrahydrotanshinone	38.75	0.36
MOL001659	Poriferasterol	43.83	0.76
MOL001771	Poriferast-5-en-3beta-ol	36.91	0.75
MOL001942	Isoimperatorin	45.46	0.23
MOL002222	Sugiol	36.11	0.28
MOL002651	Dehydrotanshinone II A	43.76	0.4
MOL002776	Baicalin	40.12	0.75
MOL000569	Digallate	61.85	0.26
MOL000006	Luteolin	36.16	0.25
MOL006824	α-Amyrin	39.51	0.76
MOL007036	5,6-Dihydroxy-7-isopropyl-1,1-dimethyl-2,3-dihydrophenanthren-4-one	33.77	0.29
MOL007041	2-Isopropyl-8-methylphenanthrene-3,4-dione	40.86	0.23
MOL007045	3α-HydroxytanshinoIIa	44.93	0.44
MOL007048	(E)-3-[2-(3,4-dihydroxyphenyl)-7-hydroxy-benzofuran-4-yl]acrylic acid	48.24	0.31
MOL007049	4-Methylenemiltirone	34.35	0.23
MOL007050	2-(4-Hydroxy-3-methoxyphenyl)-5-(3-hydroxypropyl)-7-methoxy-3-benzofurancarboxaldehyde	62.78	0.4
MOL007051	6-*o*-Syringyl-8-o-acetyl shanzhiside methyl ester	46.69	0.71
MOL007058	Formyltanshinone	73.44	0.42
MOL007059	3-Beta-hydroxymethyllenetanshiquinone	32.16	0.41
MOL007061	Methylenetanshinquinone	37.07	0.36
MOL007063	Przewalskin a	37.11	0.65
MOL007064	Przewalskin b	110.32	0.44
MOL007068	Przewaquinone B	62.24	0.41
MOL007069	Przewaquinone c	55.74	0.4
MOL007070	(6S,7R)-6,7-dihydroxy-1,6-dimethyl-8,9-dihydro-7H-naphtho[8,7-g]benzofuran-10,11-dione	41.31	0.45
MOL007071	Przewaquinone f	40.31	0.46
MOL007077	Sclareol	43.67	0.21
MOL007079	Tanshinaldehyde	52.47	0.45
MOL007081	Danshenol B	57.95	0.56
MOL007082	Danshenol A	56.97	0.52
MOL007085	Salvilenone	30.38	0.38
MOL007088	Cryptotanshinone	52.34	0.4
MOL007093	Dan-shexinkum d	38.88	0.55
MOL007094	Danshenspiroketallactone	50.43	0.31
MOL007098	Deoxyneocryptotanshinone	49.4	0.29
MOL007100	Dihydrotanshinlactone	38.68	0.32
MOL007101	Dihydrotanshinone I	45.04	0.36
MOL007105	Epidanshenspiroketallactone	68.27	0.31
MOL007107	C09092	36.07	0.25
MOL007108	Isocryptotanshi-none	54.98	0.39
MOL007111	Isotanshinone II	49.92	0.4
MOL007115	Manool	45.04	0.2
MOL007118	Microstegiol	39.61	0.28
MOL007119	Miltionone I	49.68	0.32
MOL007120	Miltionone II	71.03	0.44
MOL007121	Miltipolone	36.56	0.37
MOL007122	Miltirone	38.76	0.25
MOL007123	Miltirone II	44.95	0.24
MOL007124	Neocryptotanshinone ii	39.46	0.23
MOL007125	Neocryptotanshinone	52.49	0.32
MOL007127	1-Methyl-8,9-dihydro-7H-naphtho[5,6-g]benzofuran-6,10,11-trione	34.72	0.37
MOL007130	Prolithospermic acid	64.37	0.31
MOL007132	(2R)-3-(3,4-dihydroxyphenyl)-2-[(Z)-3-(3,4-dihydroxyphenyl)acryloyl]oxy-propionic acid	109.38	0.35
MOL007140	(Z)-3-[2-[(E)-2-(3,4-dihydroxyphenyl)vinyl]-3,4-dihydroxy-phenyl]acrylic acid	88.54	0.26
MOL007141	Salvianolic acid g	45.56	0.61
MOL007142	Salvianolic acid j	43.38	0.72
MOL007143	Salvilenone I	32.43	0.23
MOL007145	Salviolone	31.72	0.24
MOL007149	NSC 122421	34.49	0.28
MOL007150	(6S)-6-Hydroxy-1-methyl-6-methylol-8,9-dihydro-7H-naphtho[8,7-g]benzofuran-10,11-quinone	75.39	0.46
MOL007151	Tanshindiol B	42.67	0.45
MOL007152	Przewaquinone E	42.85	0.45
MOL007154	Tanshinone iia	49.89	0.4
MOL007155	(6S)-6-(hydroxymethyl)-1,6-dimethyl-8,9-dihydro-7H-naphtho[8,7-g]benzofuran-10,11-dione	65.26	0.45
MOL007156	Tanshinone VI	45.64	0.3

**Fig. 2:**
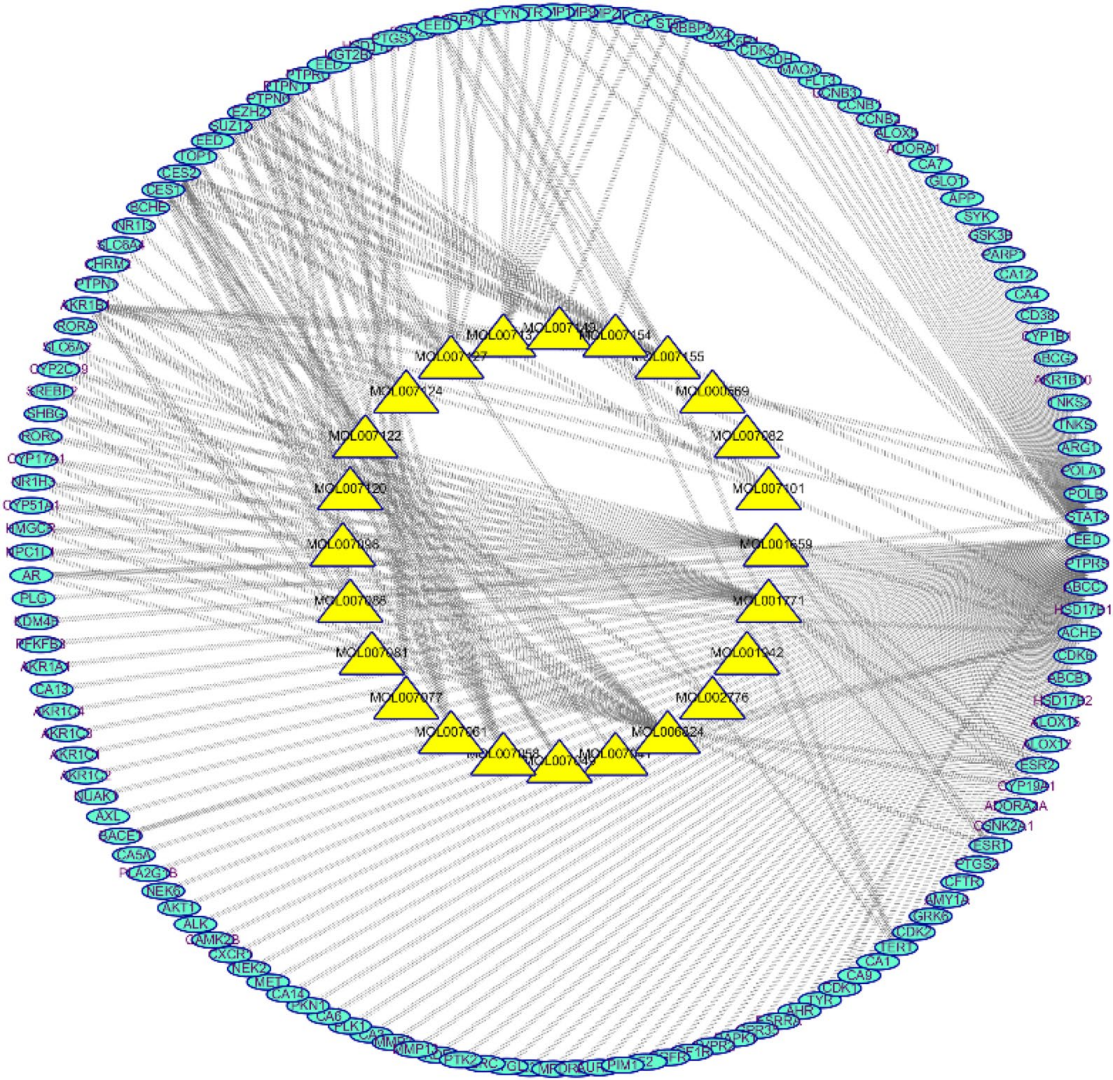
The C-T network that consists of 24 nodes and 247 targets. Yellow and blue nodes denote the compounds and targets, respectively

**Fig. 3:**
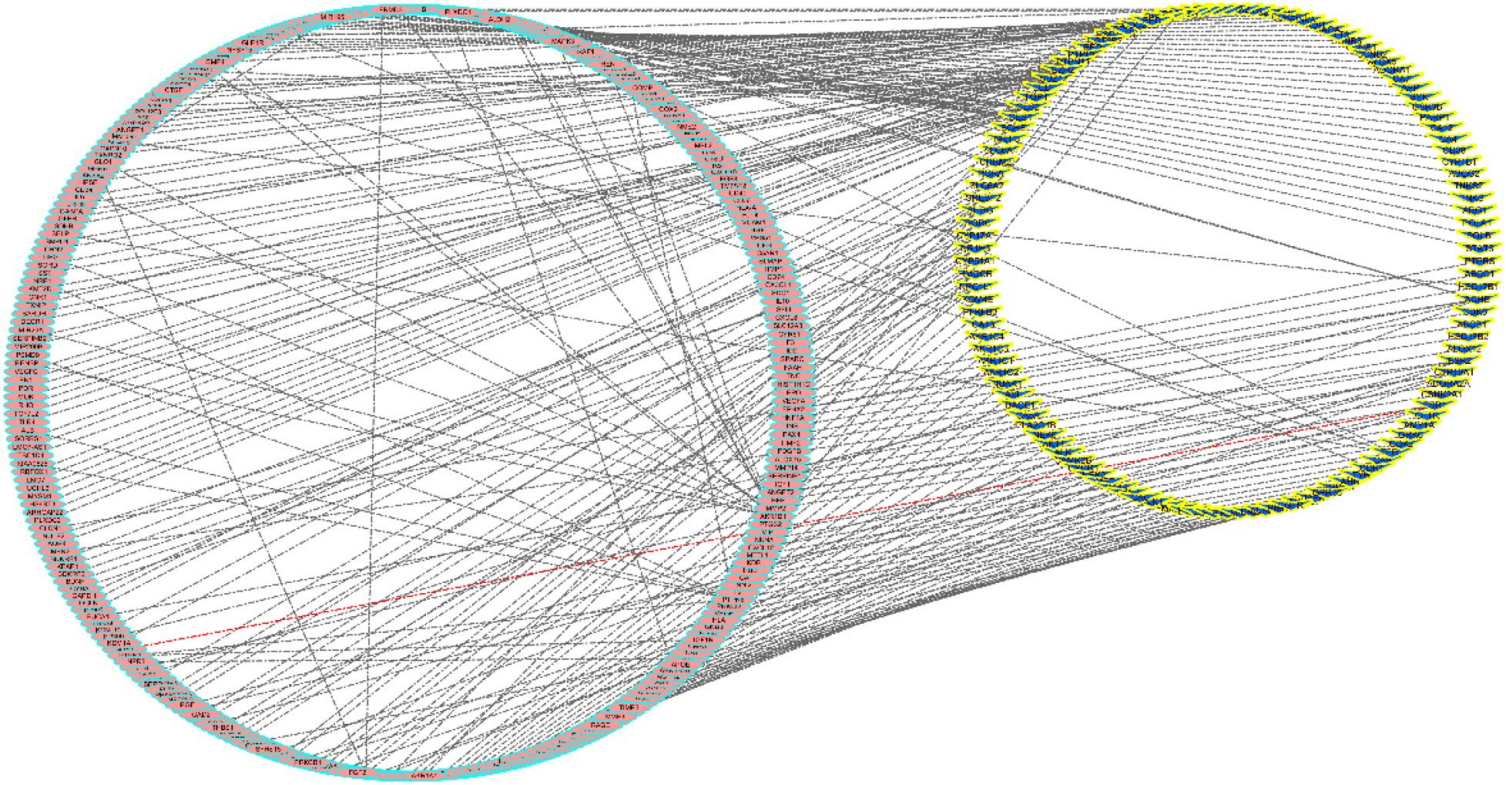
The T-D network that consists of 287 nodes and 247 targets. Pink and yellow nodes denote the diseases and targets, respectively

The target data on DR from OMIM, Drugbank, GAD, and DisGeNet was integrated. Eighteen targets that matched the related targets of *Radix Salviae* on DR were collected as related targets for the effect of *Radix Salviae* on DR (Figs. 2 and 3).

**Fig. 4:**
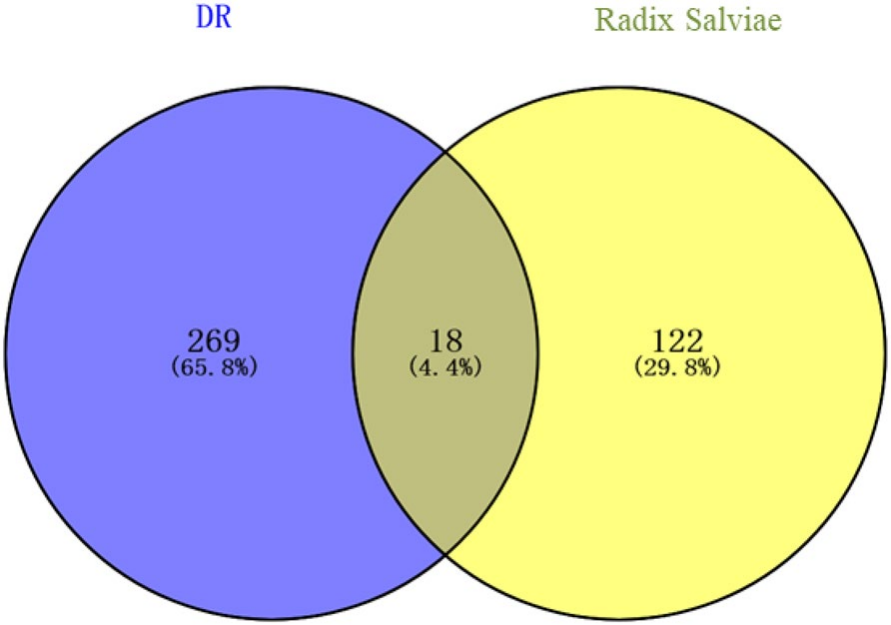
The 18 matching targets of the related targets in *Radix Salviae* on DR. *DR* diabetic retinopathy

**Fig. 5:**
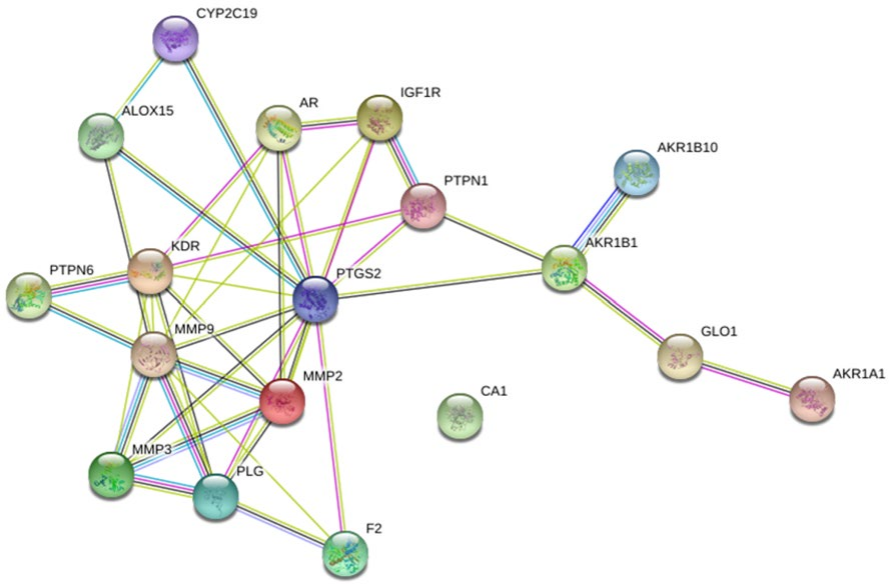
PPI network of 18 nodes and 40 edges established in the String database

In the String database, the PPI network of the 18 targets was established. The details are shown in Fig. 4. There were 18 nodes and 40 edges in total. The topological feature analysis of the PPI selected targets used median values to determine key targets and constructed the big hub nodes as the main targets that may cause the effect of *Radix Salviae* on DR, based on “degree” and “closeness certainty”. The threshold values were degree ≥ 4.8 and closeness ≥ 0.51 and the results settled at 18 hub nodes and 40 edges. The details are shown in Fig. 5, which includes prostaglandin-endoperoxide synthase 2 (PTGS2) (degree = 12), matrix metallopeptidase 9 (MMP9) (degree = 10), vascular endothelial growth factor receptor 2 (KDR) (degree = 8), matrix metallopeptidase 2 (MMP2) (degree = 7), plasminogen (PLG) (degree = 6), androgen receptor (degree = 5), matrix metallopeptidase 3 (MMP3) (degree = 5), and insulin-like growth factor 1 (IGF-1) receptor (degree = 5).

### GO biological process and KEGG pathway enrichment analysis

DAVID v6.8 was used for enrichment analysis of the 18 targets. The screening threshold was P < 0.01 and 30 GO items were retrieved. We selected 10 KEGG pathways for analysis.

### GO biological process enrichment analysis

**Fig. 6:**
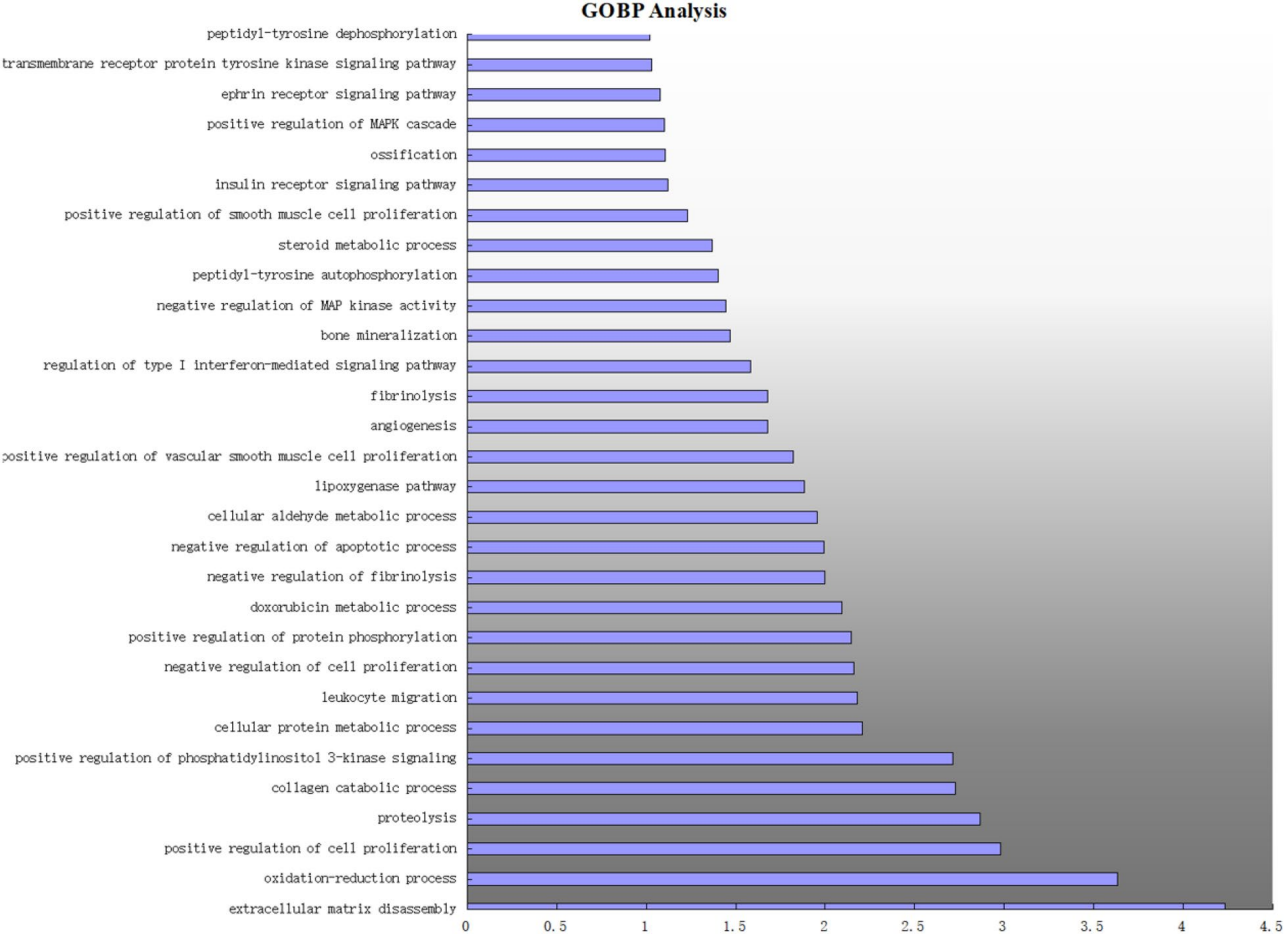
Main 30 GO biological process by major hubs from the DAVID database

The 30 biological processes were mainly involved in angiogenesis, protein metabolism, inflammatory response, apoptosis, and cell proliferation. The details are shown in Fig. 6. The processes were, in the aspect of angiogenesis: angiogenesis (GO:0001525) and positive regulation of vascular smooth muscle cell proliferation (GO:1904707); in the aspect of protein metabolism: proteolysis (GO:0006508), collagen catabolic process (GO:0030574), cellular protein metabolic process (GO:0044267), negative regulation of fibrinolysis (GO:0051918), and fibrinolysis (GO:0042730); in the aspect of inflammatory response: regulation of type I interferon-mediated signaling pathway (GO:0060338), doxorubicin metabolic process (GO:0044598), and oxidation–reduction process (GO:0055114); in the aspect of apoptosis: negative regulation of apoptotic process (GO:0043066); and, in the aspect of cell proliferation: positive regulation of cell proliferation (GO:0008284), negative regulation of cell proliferation (GO:0008285), positive regulation of vascular smooth muscle cell proliferation (GO:1904707), and positive regulation of smooth muscle cell proliferation (GO:0048661). Based on these five main aspects, a complex multi-path synergetic effect may be the cause of the effect of *Radix Salviae* on DR.

#### KEGG pathway enrichment analysis

**Fig. 7:**
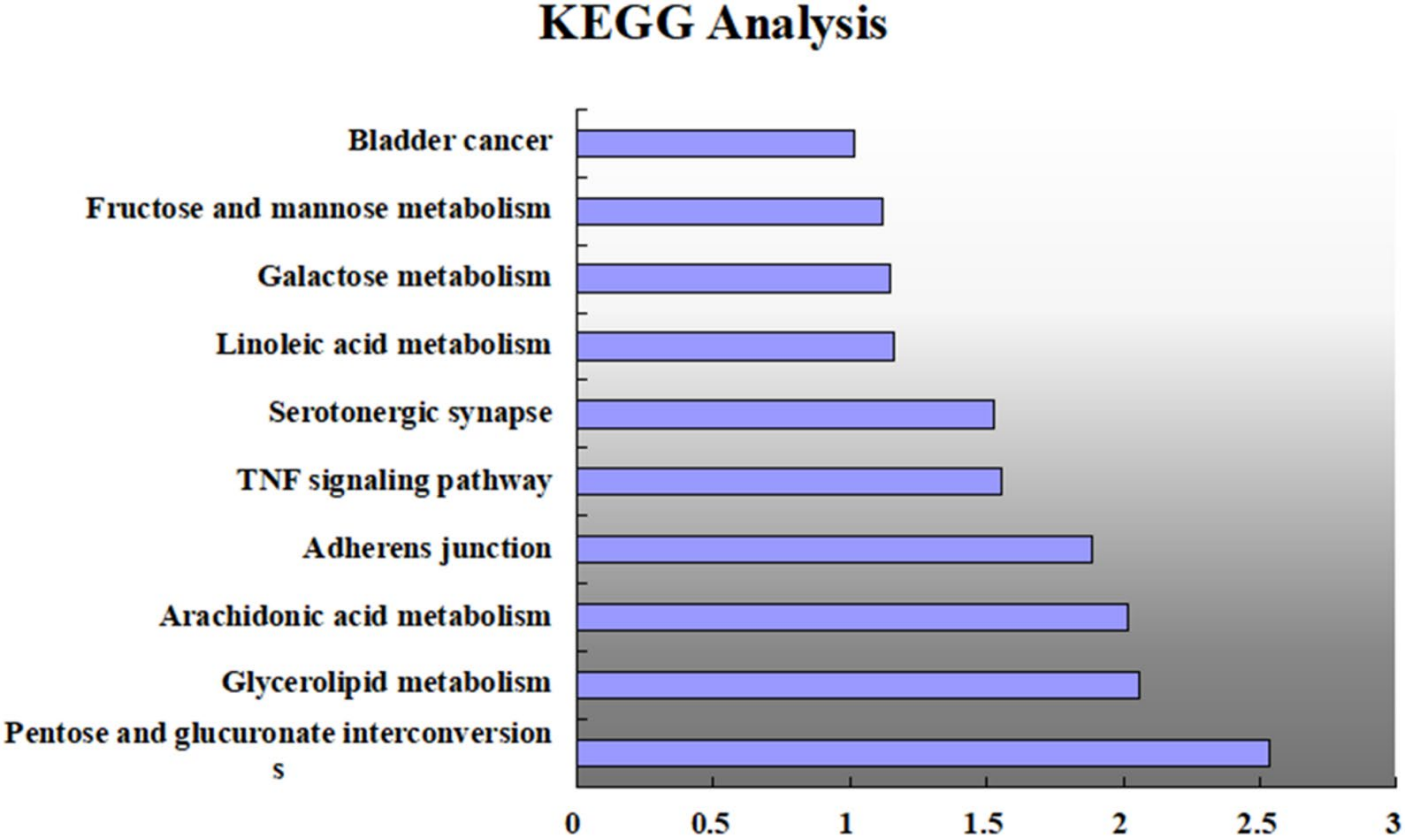
The main 10 pathways enriched by major hubs from the DAVID database

We conducted KEGG pathway enrichment analysis on 18 targets and screened 10 signaling pathways: pentose and glucuronate interconversions (hsa00040), glycerol-lipid metabolism (hsa00561), arachidonic acid metabolism (hsa00590), adherens junction (hsa04520), tumor necrosis factor signaling pathway (hsa04668), serotonergic synapse (hsa04726), linoleic acid metabolism (hsa00591), galactose metabolism (hsa00052), and fructose and mannose metabolism (hsa00051),and bladder cancer (hsa05219). The details are shown in Fig. 7.

## Discussion

We discovered that *Radix Salviae* significantly alleviated the symptoms of DR and believe that its impact may be related to its potential function. Hence, we explored the potential mechanism by the network pharmacology approach. DR is one of the most common and serious microvascular complications in type 2 diabetes patients. The basic pathological changes of DR include the loss of perithelial cell selectivity, thickening of the basement membrane, formation of microangioma, proliferation of endothelial cells, and neovascularization [32].

### Angiogenesis

#### We discovered that the anti-angiogenesis effect of *Radix Salviae* may be a factor in its treatment of DR

During hyperglycemia, the activity of MMP9 is increased and the degradation of the basement membrane is accelerated, which degrades the cell matrix in the basement membrane, loosens the cell structure, and provides nutrients and growth space for the formation of new blood vessels [33]. KDR modulates angiogenic responses such as endothelial cell migration and proliferation. VEGF acts through high-affinity receptors and some consist of KDR [34].

VEGF is of major importance in proliferative DR [35] and can induce vascular abnormalities including vascular leakage and neovascularisation [36]. The VEGF and VEGFR system plays a major role in retinal neovacularization [37]. The inhibition of the binding of VEGF to its receptor can reduce neovacularization [38]. The role of VEGF in the pathogenesis of diabetic macular edema (DME) has been widely recognized. The intravitreal injection of anti-VEGF drugs has achieved good effects in the improvement of vision and reduction of macular edema and has become the initial therapy for DME [39]. Anti-VEGF treatment of proliferative DR may be superior to pan-retinal photocoagulation (PRP) and may delay or reduce the need for vitrectomy in vitreous hemorrhage cases when PRP is not possible [6]. Anti-VEGF therapeutics, including bevacizumab, ranibizumab, and aboxicept, are effective treatments for central macular edema [40].

PLG is a blood zymogen that is activated by proteolysis and converted to plasmin and angiostatin. Plasmin dissolves fibrin in blood clots and is an important protease in many other cellular processes, whereas angiostatin inhibits angiogenesis. Defects in *PLG* are likely a cause of thrombophilia. Tissue plasminogen activator (t-PA) and its inhibitor (PAI), participate in neovascularization, particularly in VEGF expression. Vascular tissue formation and new matrix component production is the basis of vascular proliferation. t-PA plays a major role in endothelial cell matrix degradation, which is an important precondition for endothelial cell proliferation and migration [41]. In addition, the expression of both t-PA and PAI is significantly correlated with VEGF expression [42].

IGF-1 stimulates growth, differentiation, and metabolism in a variety of cell types and plays a crucial role in both embryonic and postnatal growth. IGF-I is synthesized by the liver and acts on the tyrosine-kinase receptor (IGF-1R). IGF-1 expression is preserved in many tissues, including the retina [43]. Several retinal cell types, such as endothelial and retinal pigment epithelium cells, express both IGF-1 and its receptor. Injected IGF-1 induces retinal neovascularization and blood-retinal barrier breakdown in several in vitro studies [44].

### Apoptosis

#### We discovered that the inhibition of apoptosis induced by *Radix Salviae* may be a factor in its treatment of DR

In the pathogenesis of DR, retinal microvascular cells (pericytes and endothelial cells) and other cells, including glial cells and neuronal cells, are lost selectively via apoptosis [45]. Apart from this, the accelerated loss of capillary cells may increase oxidative stress and inflammatory mediators [46]. In addition, the high level of manganese superoxide in the mitochondria has an important role in DR [47].

MMP, a member of the proteinase family, regulates major biological functions, including tissue repair and cell signaling. Among the MMPs, MMP2 is the most ubiquitous [48]. Activated MMP2 in the mitochondria results in the accelerated apoptosis of retinal capillary cells in diabetes, which damages the retinal mitochondria by modulating Hsp60 and connexin 43 and allows cytochrome c to leak out and activate apoptotic machinery [49].

### Inflammatory response

#### We discovered that the induction of the inflammatory response by *Radix Salviae* may be a factor in its treatment of DR

Prostaglandin-endoperoxide synthase, also known as cyclooxygenase, is the key enzyme in prostaglandin biosynthesis and acts as both a dioxygenase and peroxidase. PTGS2, which is often called cyclooxygenase 2 (COX-2), is responsible for the prostanoid biosynthesis involved in inflammation and mitogenesis. In the plasma membrane, hyperglycemia activates aquaporin-1, which can sense osmolarity changes, and an “osmosignaling” pathway, which involves the transcription factor tonicity enhancer binding protein, which transmits the signal towards effector regulatory sites in the nuclei. This further promotes the expression of pro-inflammatory genes such as adhesion molecules and COX-2 [50].

### Radix Salviae

**Fig. 8:**
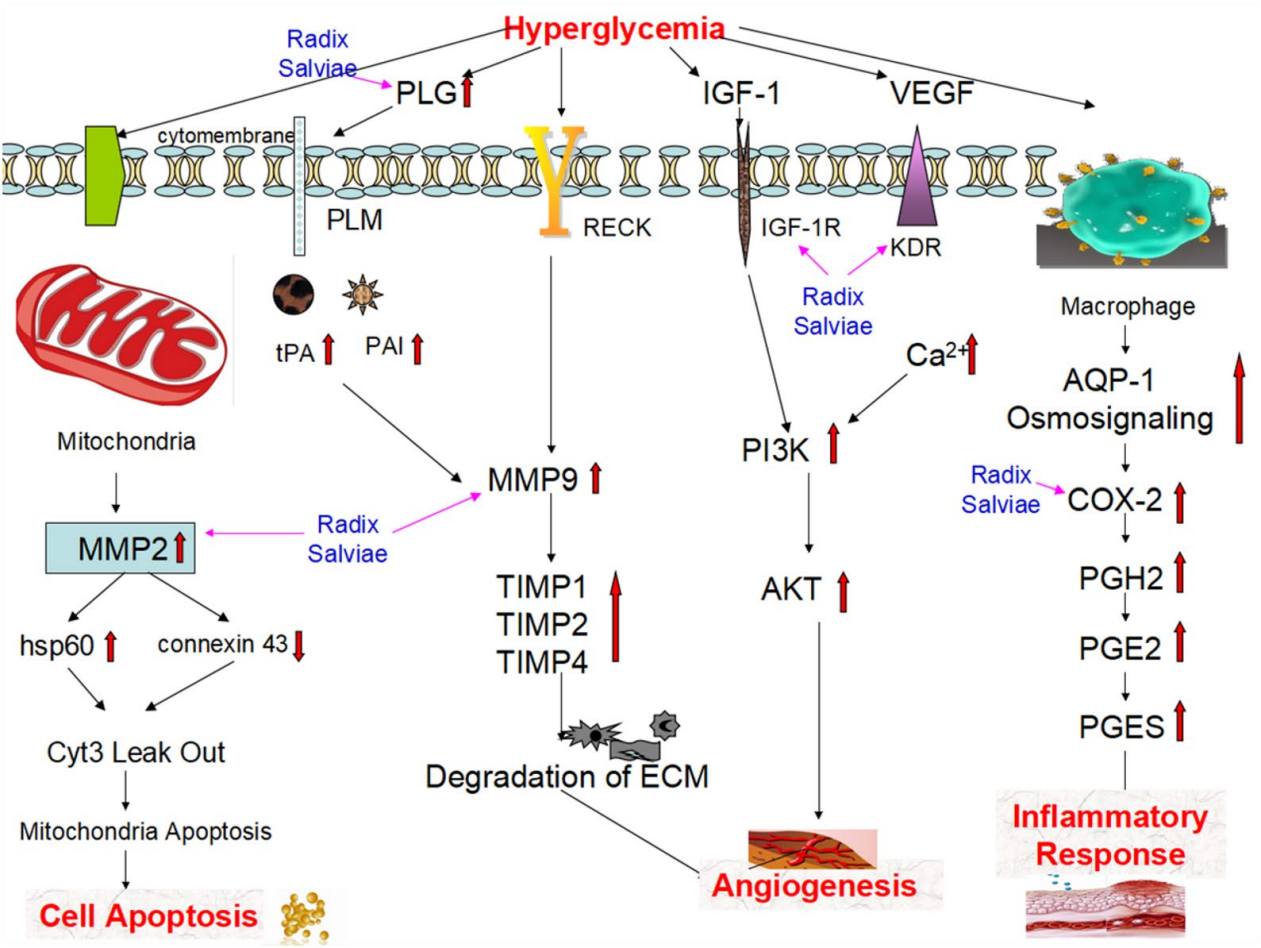
Illustration of the crucial biological processes caused by putative targets and known therapeutic targets for DR. *MMP2* matrix metallopeptidase 2, *hsp60* heat shock protein 60, *Cyt3* cytochrome 3, *PLG* plasminogen, *PLM* plasmin, *tPA* tissue plasminogen activator, *PAI* plasminogen activator inhibitor, *RECK* reversion-inducing cysteine-rich protein with Kazal motifs, *MMP9* matrix metallopeptidase 9, *TIMP 1* tissue inhibitor of metalloproteinases 1, *TIMP 2* tissue inhibitor of metalloproteinases 2, *TIMP 4* tissue inhibitor of metalloproteinases 4, *ECM* extracellular matrix, *IGF-1* insulin-like growth factor-1, *IGF-1R* insulin-like growth factor-1 receptor, *PI3K* phosphatidylinositol 3-kinase, *AKT* serine-threonine kinase, *VEGF* vascular endothelial growth factor, *KDR* vascular endothelial growth factor receptor 2, *AQP-1* aquaporin-1, *COX-2* cyclooxygenase-2, *PGH2* prostaglandin H2, *PGE2* prostaglandin E2, *PGES* prostaglandin E2 synthase

*Radix Salviae* is one of the most popular Chinese herbs and has been used for centuries for the management of cardiovascular and cerebrovascular diseases [51]. There are several studies that demonstrate that *Radix Salviae* can effectively reduce apoptosis, cell proliferation, and neovascularization. Liu et al. [52] believed that Danhong injection (DHI) could induce the expression of insulin receptor substrate 1, fibroblast growth factor 21, and peroxisome proliferator-activated receptor gamma in the liver and peripheral tissues, which can increase insulin sensitivity. Furthermore, the induction of genes involved in lipolysis, fatty acid oxidation, and mitochondrial biogenesis suggests that DHI may enhance energy metabolism. Moreover, DHI inhibits CRISPR-associated gene 3, MMP2, and MMP9 expression and the formation of acellular capillaries in retinas; thus, DHI can prevent diabetes-induced apoptosis and protect retinas against diabetes-induced damage. Salvianolic acid B (Sal B), one of the major water-soluble compounds isolated from *Radix Salviae*, can inhibit high glucose-induced cell proliferation by releasing the cell from G1 phase arrest and delaying S phase progression in human mesangial cells. Sal B can decrease the secretion of high glucose-induced MMP2 and MMP9, which is partly mediated by blocking NF-κB activation [53]. Sal A has a wide range of pharmacological effects, such as anti-inflammatory, antioxidant, and anti-fibrotic properties [51, 54, 55]. In addition, the Chinese herbal compound *Radix Salviae* is widely used in clinical practice. A recent meta-analysis shows that the herb *Salvia miltiorrhiza* (*Radix Salviae* dripping pill, CDDP) can protect endothelial diastolic function, vision, and visual acuity, improve microvascular structure, and improve retinal microcirculation. Therefore, *Radix Salviae* can safely and effectively delay the progression of DR and loss of vision, providing a new treatment for DR (Fig. 8).

However, there are some limitations in the use of network pharmacological methods to predict active ingredients and potential mechanisms. (i) The screened active ingredients may be inconsistent with the actual absorbed components in the blood of patients with DR; (ii) it may be difficult to distinguish the inhibitory effect from the target activation effect; (iii) the predicted results may be affected by possible deviation in highly studied pathways and functions; and (iv) the interaction relationships between the nodes in the network construction methods are still unclear. Therefore, further experimental verification of the potential active ingredients is needed to verify this theoretical prediction.

## Conclusions

This study used a scientific approach to decipher the pharmacological mechanisms of *Radix Salviae* in the treatment of DR. We discovered that the effects may be associated with anti-angiogenesis, the inhibition of apoptosis, and the inflammatory response. Among these crucial biological functions, eight targets were identified as key active factors involved in the related pathways. This research suggests that *Radix Salviae* can alleviate DR via the molecular mechanisms predicted by network pharmacology and that the network pharmacology approach can be an effective tool to reveal the mechanisms of TCM. However, to improve the reliability of the results, further experimental experiments are needed to validate these results.

## Data Availability

The data and materials generated or analyzed during this study are available from the corresponding author on reasonable request.

## Abbreviations

DR: diabetic retinopathy
VEGF: vascular endothelial growth factor
TCM: Traditional Chinese Medicine
OB: oral bioavailability
DL: drug-likeness
GO: Gene Ontology
KEGG: Kyoto Encyclopedia of Genes and Genomes
PPI: protein–protein interaction
DAVID: Database for Annotation, Visualization and Integrated Discovery
C-T: compound-target
T-T: target-target
T-P: target-pathway
PTGS2: prostaglandin-endoperoxide synthase 2
MMP3: matrix metalloprotease 3
MMP2: matrix metallopeptidase 2
PLG: plasminogen
PLM: plasmin
tPA: tissue plasminogen activator
PAI: plasminogen activator inhibitor
MMP9: matrix metallopeptidase 9
IGF-1: insulin-like growth factor-1
IGF-1R: insulin-like growth factor-1 receptor
KDR: vascular endothelial growth factor receptor 2
COX-2: cyclooxygenase-2
PRP: pan-retinal photocoagulation
DHI: Danhong injection

## References

[1] American Academy of Ophthalmology Retina/Vitreous Panel. Preferred Practice Pattern^®^Guidelines. Diabetic retinopathy. San Francisco, CA: American Academy of Ophthalmology; 2017. http://www.aao.org/ppp. Accessed 15 Aug 2019.

[2] Leasher Janet L., Bourne Rupert R.A., Flaxman Seth R., Jonas Jost B., Keeffe Jill, Naidoo Novin, Pesudovs Konrad, Price Holly, White Richard A., Wong Tien Y., Resnikoff Serge, Taylor Hugh R. (2016). Erratum. Global Estimates on the Number of People Blind or Visually Impaired by Diabetic Retinopathy: A Meta-analysis From 1990–2010. Diabetes Care 2016;39:1643–1649. Diabetes Care.

[3] Flaxman SR, Bourne RRA, Resnikoff S (2017). Global causes of blindness and distance vision impairment 1990–2020: a systematic review and meta-analysis. Lancet Glob Health..

[4] Rees G, Xie J, Fenwick EK (2016). Association between diabetes-related eye complications and symptoms of anxiety and depression. JAMA Ophthalmol..

[5] Kramer CK, Rodrigues TC, Canani LH, Gross JL, Azevedo MJ (2011). Diabetic retinopathy predicts all-cause mortality and cardiovascular events in both type 1 and 2 diabetes: meta-analysis of observational studies. Diabetes Care.

[6] Zhao Y, Singh RP (2018). The role of anti-vascular endothelial growth factor (anti-VEGF) in the management of proliferative diabetic retinopathy. Drugs Context..

[7] Whitcup SM, Cidlowski JA, Csaky KG, Ambati J (2018). Pharmacology of corticosteroids for diabetic macular edema. Invest Ophthalmol Vis Sci.

[8] Li S, Xutian S (2016). New development in Traditional Chinese Medicine: symbolism-digit therapy as a special naturopathic treatment. Am J Chin Med.

[9] Li S, Zhang B, Zhang N (2011). Network target for screening synergistic drug combinations with application to Traditional Chinese Medicine. BMC Syst Biol.

[10] Fitzgerald JB, Schoeberl B, Nielsen UB, Sorger PK (2006). Systems biology and combination therapy in the quest for clinical efficacy. Nat Chem Biol.

[11] Csermely P, Agoston V, Pongor S (2005). The efficiency of multi-target drugs: the network approach might help drug design. Trends Pharmacol Sci.

[12] Zheng J, Wu M, Wang H (2018). Network pharmacology to unveil the biological basis of health-strengthening herbal medicine in cancer treatment. Cancers..

[13] Xu L, Zhang Y, Zhang P (2019). Integrated metabolomics and network pharmacology strategy-driven active traditional chinese medicine ingredients discovery for the alleviation of cisplatin nephrotoxicity. Chem Res Toxicol.

[14] Liu J, Li Y, Zhang Y (2019). A Network Pharmacology Approach to Explore the Mechanisms of Qishen Granules in Heart Failure. Med Sci Monit Int Med J Exp Clin Res.

[15] Dong Z, Tao X, Fu X, Wang H, Wang D, Zhang T (2012). Protective effects of Purendan superfine powder on retinal neuron apoptosis in a rat model of type 2 diabetes mellitus. Neural Regen Res..

[16] Lian F, Wu L, Tian J (2015). The effectiveness and safety of a danshen-containing Chinese herbal medicine for diabetic retinopathy: a randomized, double-blind, placebo-controlled multicenter clinical trial. J Ethnopharmacol.

[17] Hopkins AL (2008). Network pharmacology: the next paradigm in drug discovery. Nat Chem Biol.

[18] Zhang Y, Bai M, Zhang B (2015). Uncovering pharmacological mechanisms of Wu-tou decoction acting on rheumatoid arthritis through systems approaches: drug-target prediction, network analysis and experimental validation. Sci Rep..

[19] Ru J, Li P, Wang J (2014). TCMSP: a database of systems pharmacology for drug discovery from herbal medicines. J Cheminform..

[20] Xu X, Zhang W, Huang C (2012). A novel chemometric method for the prediction of human oral bioavailability. Int J Mol Sci.

[21] Tao W, Xu X, Wang X (2013). Network pharmacology-based prediction of the active ingredients and potential targets of Chinese herbal Radix Curcumae formula for application to cardiovascular disease. J Ethnopharmacol.

[22] Gfeller D, Michielin O, Zoete V (2013). Shaping the interaction landscape of bioactive molecules. Bioinformatics.

[23] Amberger JS, Hamosh A (2017). Searching online mendelian inheritance in man (OMIM): a knowledgebase of human genes and genetic phenotypes. Curr Protoc Bioinformatics..

[24] Pinero J, Bravo A, Queralt-Rosinach N (2017). DisGeNET: a comprehensive platform integrating information on human disease-associated genes and variants. Nucleic Acids Res.

[25] Wang H, Liu X, Tao Y (2019). Automatic human-like mining and constructing reliable genetic association database with deep reinforcement learning. Pac Symp Biocomput..

[26] Szklarczyk D, Morris JH, Cook H (2017). The STRING database in 2017: quality-controlled protein-protein association networks, made broadly accessible. Nucleic Acids Res.

[27] Shannon P, Markiel A, Ozier O (2003). Cytoscape: a software environment for integrated models of biomolecular interaction networks. Genome Res.

[28] Missiuro PV, Liu K, Zou L (2009). Information flow analysis of interactome networks. PLoS Comput Biol.

[29] Tang Y, Li M, Wang J, Pan Y, Wu FX (2015). CytoNCA: a cytoscape plugin for centrality analysis and evaluation of protein interaction networks. Biosystems..

[30] da Huang W, Sherman BT, Lempicki RA (2009). Systematic and integrative analysis of large gene lists using DAVID bioinformatics resources. Nat Protoc.

[31] Chen L, Zhang YH, Wang S, Zhang Y, Huang T, Cai YD (2017). Prediction and analysis of essential genes using the enrichments of gene ontology and KEGG pathways. PLoS ONE.

[32] Lechner J, O’Leary OE, Stitt AW (2017). The pathology associated with diabetic retinopathy. Vision Res.

[33] Herszenyi L, Hritz I, Pregun I (2007). Alterations of glutathione S-transferase and matrix metalloproteinase-9 expressions are early events in esophageal carcinogenesis. World J Gastroenterol.

[34] Li F, Huang J, Ji D (2017). Azithromycin effectively inhibits tumor angiogenesis by suppressing vascular endothelial growth factor receptor 2-mediated signaling pathways in lung cancer. Oncol Lett..

[35] Thomas KA (1996). Vascular endothelial growth factor, a potent and selective angiogenic agent. J Biol Chem.

[36] Weis SM, Cheresh DA (2005). Pathophysiological consequences of VEGF-induced vascular permeability. Nature.

[37] Adams RH, Alitalo K (2007). Molecular regulation of angiogenesis and lymphangiogenesis. Nat Rev Mol Cell Biol.

[38] Olmos LC, Sayed MS, Moraczewski AL (2016). Long-term outcomes of neovascular glaucoma treated with and without intravitreal bevacizumab. Eye (Lond)..

[39] Cai S, Yang Q, Li X, Zhang Y (2018). The efficacy and safety of aflibercept and conbercept in diabetic macular edema. Drug Des Devel Ther..

[40] Wells JA, Glassman AR, Ayala AR (2015). Aflibercept, bevacizumab, or ranibizumab for diabetic macular edema. N Engl J Med.

[41] Singh R, Ramasamy K, Abraham C, Gupta V, Gupta A (2008). Diabetic retinopathy: an update. Indian J Ophthalmol.

[42] Wu SL, Zhan DM, Xi SH, He XL (2014). Roles of tissue plasminogen activator and its inhibitor in proliferative diabetic retinopathy. Int J Ophthalmol..

[43] Ruiz de Almodovar C, Luttun A, Carmeliet P (2006). An SDF-1 trap for myeloid cells stimulates angiogenesis. Cell..

[44] Le Roith D, Bondy C, Yakar S, Liu JL, Butler A (2001). The somatomedin hypothesis: 2001. Endocr Rev.

[45] Poulaki V, Joussen AM, Mitsiades N, Mitsiades CS, Iliaki EF, Adamis AP (2004). Insulin-like growth factor-I plays a pathogenetic role in diabetic retinopathy. Am J Pathol.

[46] Roy S, Amin S, Roy S (2016). Retinal fibrosis in diabetic retinopathy. Exp Eye Res.

[47] Shin YI, Nam KY, Lee SE (2019). Peripapillary microvasculature in patients with diabetes mellitus: an optical coherence tomography angiography study. Sci Rep..

[48] Barber AJ, Lieth E, Khin SA, Antonetti DA, Buchanan AG, Gardner TW (1998). Neural apoptosis in the retina during experimental and human diabetes. Early onset and effect of insulin. J Clin Invest..

[49] Mohammad G, Kowluru RA (2011). Novel role of mitochondrial matrix metalloproteinase-2 in the development of diabetic retinopathy. Invest Ophthalmol Vis Sci.

[50] Madonna R, Giovannelli G, Confalone P, Renna FV, Geng YJ, De Caterina R (2016). High glucose-induced hyperosmolarity contributes to COX-2 expression and angiogenesis: implications for diabetic retinopathy. Cardiovasc Diabetol..

[51] Tang MK, Ren DC, Zhang JT, Du GH (2002). Effect of salvianolic acids from Radix Salviae miltiorrhizae on regional cerebral blood flow and platelet aggregation in rats. Phytomedicine.

[52] Liu M, Pan Q, Chen Y (2015). Administration of Danhong Injection to diabetic db/db mice inhibits the development of diabetic retinopathy and nephropathy. Sci Rep..

[53] Luo P, Tan Z, Zhang Z, Li H, Mo Z (2008). Inhibitory effects of salvianolic acid B on the high glucose-induced mesangial proliferation via NF-kappaB-dependent pathway. Biol Pharm Bull.

[54] Yang LL, Li DY, Zhang YB, Zhu MY, Chen D, Xu TD (2012). Salvianolic acid A inhibits angiotensin II-induced proliferation of human umbilical vein endothelial cells by attenuating the production of ROS. Acta Pharmacol Sin.

[55] Li HY, Li Y, Yan CH, Li LN, Chen XG (2002). Inhibition of tumor growth by S-3-1, a synthetic intermediate of salvianolic acid A. J Asian Nat Prod Res.

